# Characteristics and Functions of the Rumen Microbial Community of Cattle-Yak at Different Ages

**DOI:** 10.1155/2020/3482692

**Published:** 2020-03-03

**Authors:** Yuzhu Sha, Jiang Hu, Bingang Shi, Renqing Dingkao, Jiqing Wang, Shaobin Li, Wei Zhang, Yuzhu Luo, Xiu Liu

**Affiliations:** ^1^College of Animal Science and Technology/Gansu Key Laboratory of Herbivorous Animal Biotechnology, Gansu Agricultural University, Lanzhou 730070, China; ^2^Gannan Institute of Animal Husbandry Science, Hezuo 747000, China

## Abstract

A cattle-yak, which is a hybrid between a yak (*Bos grunniens*) and cattle (*Bos taurus*), is an important livestock animal, but basic questions regarding its physiology and environmental adaptation remain unanswered. To address this issue, the present study examined the species composition and functional characteristics of rumen microorganisms in the cattle-yak of different ages (2 and 3 years old) by metagenomic analysis. We found that rumen microbial community composition was similar at the two ages. Firmicutes, Fibrobacteres, Euryarchaeota, Bacteroidetes, and Proteobacteria were the predominant phyla, with Firmicutes accounting for the highest percentage of bacteria in 2-year-old (48%) and 3-year-old (46%) animals. Bacterial species involved in lignocellulose degradation were detected in the rumen of adult cattle-yaks including *Ruminococcus flavefaciens*, *Ruminococcus albus*, *Fibrobacter succinogenes*, and *Prevotella ruminicola*, with *F. succinogenes* being the most abundant. A total of 145,489 genes were annotated according to the Carbohydrate-active Enzyme database, which identified glycoside hydrolases as the most highly represented enzyme family. Further functional annotation revealed specific microflora and genes in the adult rumen that are potentially related to plateau adaptability. These results could explain the heterosis of the cattle-yak and provide insight into mechanisms of physiologic adaptation in plateau animals.

## 1. Introduction

Ruminants have a diverse microbiota in their rumen including bacteria, fungi, archaea, protozoa, and viruses. These microorganisms can degrade the plant cell wall and fibrous substances that are converted into absorbable compounds such as proteins and volatile fatty acids (VFAs) [1, 2]. One study analyzing the rumen microbiome of Indian buffalo identified 2614 contigs encoding putative degradative enzymes [3], and another reported 42 operational taxonomic units representing the rumen bacterial community of adult buffalo [4]. Microbiome profile can vary according to the developmental stage; for instance, rumen microbial community composition in cows changes markedly from birth to the age of 2 years [5]. The rumen microbial environment of adult cattle is more stable and stricter than that of newborn calves, although it is unclear whether it also changes throughout adulthood.

A cattle-yak is the F1 hybrid between the female yak (*Bos grunniens*) and male cattle (*Bos taurus*), with the male cattle-yak being sterile. Cattle-yaks exhibit greater adaptability to harsh environments and provide more meat per weight than yak [6], along with milk, wool, fuel, and other products for communities living in the Qinghai-Tibet Plateau, making it an important livestock animal. Cattle-yaks live at high altitudes and feed mostly by grazing; they can also grow normally during the dry grass period of the Plateau when the food supply is limited. The rumen of cattle-yaks is presumed to contain microorganisms with lignocellulose-degrading capability that enable adaptation to the plateau environment [7], but few studies have investigated rumen microbiota community composition and function in these animals.

To address this issue, we carried out a metagenomic analysis of the rumen microbiome of cattle-yak at two different ages (2 and 3 years of age). Our results reveal the diversity of the rumen microbial community in ruminants and suggest potential mechanisms of adaptation to the unique plateau environment.

## 2. Materials and Methods

### 2.1. Ethics Statement

Experiments involving animals were carried out in accordance with regulations for the Administration of Affairs Concerning Experimental Animals (Ministry of Science and Technology, China; revised in June 2004). Sample collection was carried out according to the guidelines of the Ethics Committee for the Care and Use of Laboratory Animals of Gansu Agricultural University.

### 2.2. Animals and Sample Collection

Six male 2- and 3-year-old healthy cattle-yaks (yak ♀×Jersey cattle ♂, *n* = 3 each) were obtained from a farm in Gannan Tibetan Autonomous Prefecture (Gansu Province, China). Rumen samples (50 ml, containing fluid and feed particles) were collected from each animal after slaughter and immediately frozen in liquid nitrogen and stored at −80°C until use.

### 2.3. DNA Extraction and High-Throughput Sequencing

Genomic DNA was extracted from rumen fluid and purified using the TIANamp Stool DNA Kit (Tiangen Biotech, Beijing, China) according to the manufacturer's instructions and sent to Gansu Meita Biomedical Co. (Lanzhou, China) for high-throughput sequencing.

### 2.4. Bioinformatics and Statistical Analyses

Genomic DNA was sheared with a Covaris M220 focused ultrasonicator (Thermo Fisher Scientific, Waltham, MA, USA), yielding ~300 bp fragments for library construction. High-throughput sequencing was performed on the HiSeq 2000 platform (Illumina, San Diego, CA, USA). Low-quality and unidentified (N) bases were filtered using fastp software; clean reads were combined with the host reference genome sequence, and host contaminating sequences were removed by Burrows-Wheeler alignment (bwa) [8]. Megahit software was used for metagenomic contig assembly from single and mixed samples. Open reading frames (ORFs) and amino acid sequences were predicted with MetaGeneMark software, and a nonredundant gene set was obtained using CD-HIT-est [9]. We used information for each sample read and assembly results to compare nonredundant gene sets, and gene abundance was analyzed using bwa+featureCounts software.

### 2.5. Functional Annotation

Functional annotation based on Kyoto Encyclopedia of Genes and Genomes (KEGG), Evolutionary Genealogy of Genes: Nonsupervised Orthologous Groups (eggNOG), and Carbohydrate-active Enzyme (CAZy) databases was performed using Diamond, eggNOG-mapper, and KOBAS software packages.

## 3. Results

### 3.1. DNA Sequence Data and Microbial Diversity Analysis

A total of 102,395,772 and 83,452,932 unique reads were obtained from rumen samples of 2- and 3-year old cattle-yaks, respectively (Table 1). Over 98% of the original data were selected as clean reads. The GC content was higher for 3-year-old than for 2-year-old cattle-yak. The clean reads were filtered based on the host genome, yielding filtered host reads that constituted a total of 0.8 GB for both ages, with GC content being higher in the older animals. Contigs for single and mixed samples were combined and interrupted from N junctions to obtain sequences containing no N, and fragments < 500 bp were removed by filtering to obtain a final assembly of 1,365,143 scaffolds constituting ~1369 Mbp (Table 2). Based on the mixed-assembly ORF prediction results, CD-HIT software was used to obtain a nonredundant gene catalog. A total of 1,281,665 nonredundant genes and ~815 Mbp nonredundant gene sequences were predicted, which had an average length of 636.64 bp.

### 3.2. Analysis of Rumen Microbial Community Composition

**Figure 1:**
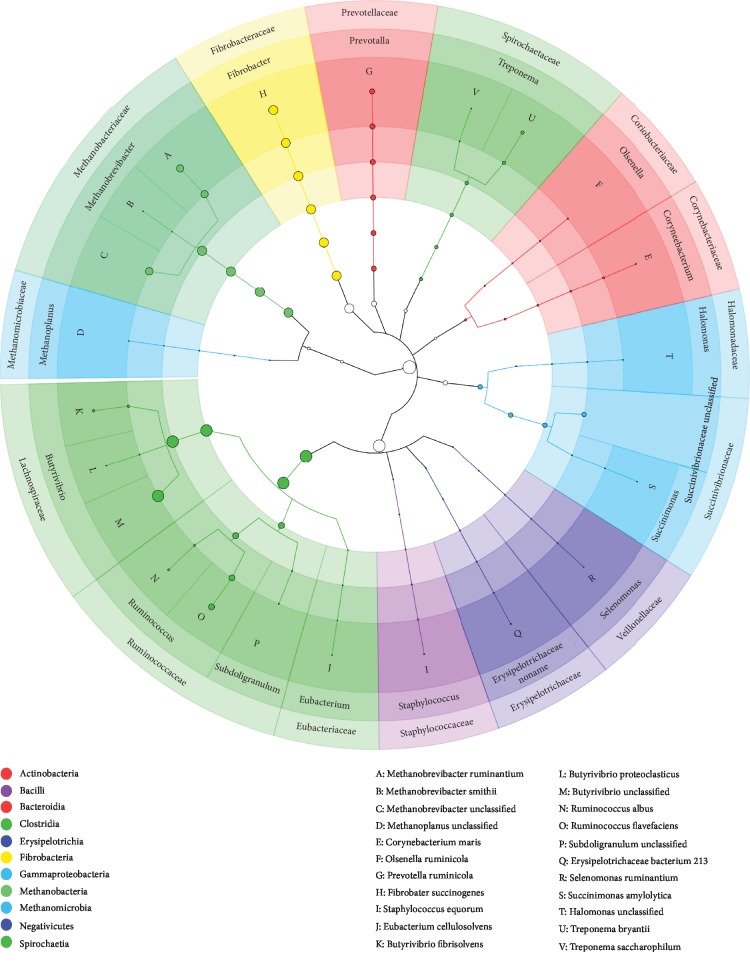
Cladogram of bacterial species in the rumen of cattle-yak.

Microbial abundance in the rumen of 2- and 3-year-old cattle-yaks was analyzed with metaphin2 software according to taxonomic classification (Figure 1). At the kingdom level, bacteria and archaea were the most highly represented taxa, with the former being predominant at 2 and 3 years of age (bacteria: 85% and 90% and archaea: 15% and 10%, respectively) (Additional file ([Supplementary-material supplementary-material-1])).

**Figure 2:**
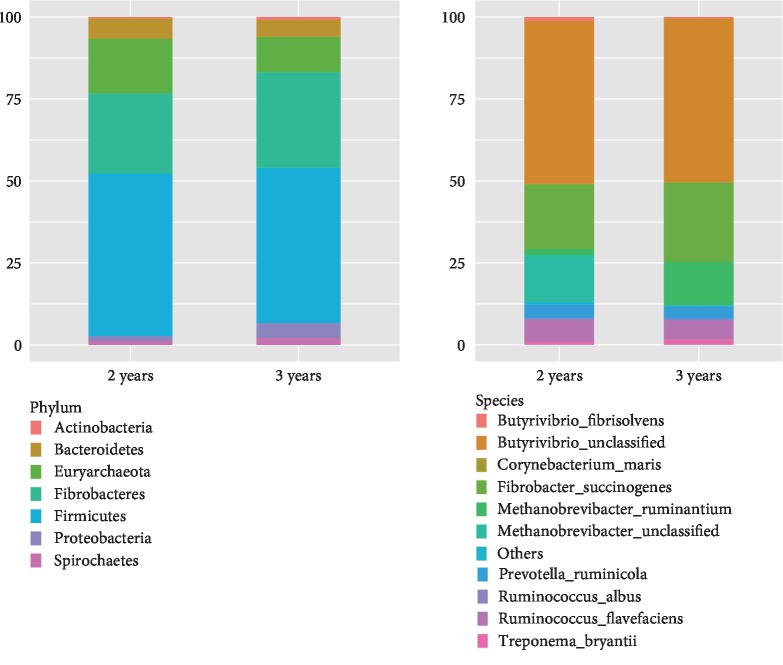
Rumen microbiota community composition at the phylum and species levels in 2- and 3-year-old cattle-yaks.

At the phylum level, the rank order of abundance was Firmicutes>Fibrobacteres>Euryarchaeota>Bacteroidetes>Proteobacteria at both ages. The abundance of Firmicutes was 48% at 2 years and 46% at 3 years of age (Figure 2). With the exception of Proteobacteria, there were no differences in the abundance of the other phyla between the two groups. In 2-year-old cattle-yaks, Proteobacteria represented 1% of total bacteria compared to 4% in 3-year-old samples (*P* < 0.001). At the species level, *Butyrivibrio* (unclassified) was the most highly represented taxon at both ages (8%), followed by *Fibrobacter succinogenes*. Additionally, multiple species related to lignocellulose degradation were detected including *Ruminococcus flavefaciens*, *Ruminococcus albus*, *F. succinogenes*, and *Prevotella ruminicola*, which were present in similar numbers in the two groups. In contrast, *Methanobrevibacter ruminantium* was more abundant in 3-year-old (2%) as compared to 2-year-old (0.3%) cattle-yaks, whereas *Methanobrevibacter* (unclassified) was not detected in the older animals.

### 3.3. Annotation of Gene Function

Functional annotation was performed for 1,281,665 nonredundant genes based on the KEGG, eggNOG, and CAZy functional databases; 62.85% of the genes were annotated to eggNOG, and 11.35% were annotated to the CAZy database (Table 3).

#### 3.3.1. CAZy Functional Annotation

**Figure 3:**
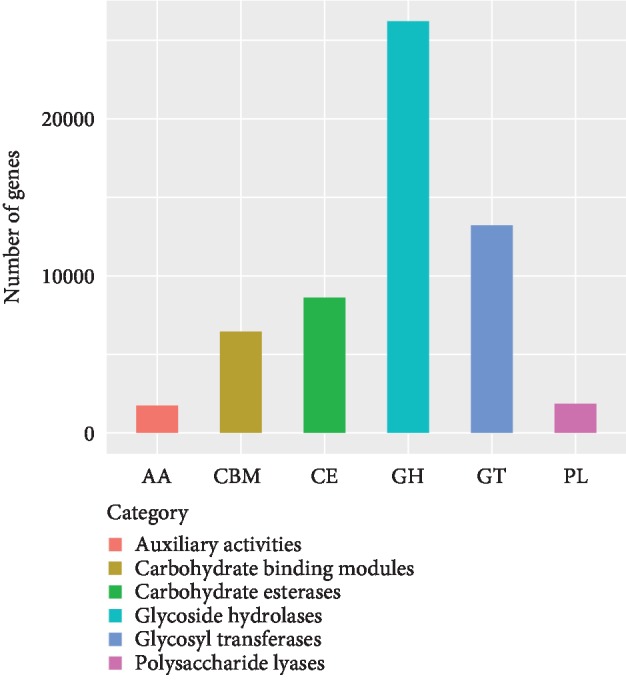
CAZy functional annotation of identified genes.

A total of 145,489 genes were aligned to the CAZy database. Glycoside hydrolase (GH) genes were the most highly represented (26,207), followed by glycosyltransferases (GTs; 13,218), carbohydrate esterases (CEs; 8615), and carbohydrate-binding modules (CBMs; 6452) (Figure 3). On the other hand, there were few genes associated with auxiliary activities (AA) and encoding polysaccharide lyases (PL) (1746 and 1863, respectively). The GH genes were distributed in various gene families, with most (>1500) belonging to the GH2, GH43, GH13, and GH5 families and GH47 and GH96 families accounting for only one gene each (Additional file). There were over 2000 genes belonging to the GT2, GT4, GT27, GT5, and GT41 families. CE1, CE7, CE10, CBM6, CBM32, and CBM50 families were the most highly represented among CE and CBM genes. In addition, 29 cohesin and 415 docker genes were identified in the rumen of cattle-yaks. The functions associated with CAZymes were similar in the two age groups, but there were minor differences: except for GTs, the other five types of CAZymes were more highly expressed in the older animals (Additional file, [Supplementary-material supplementary-material-1]).

#### 3.3.2. eggNOG Functional Annotation

**Figure 4:**
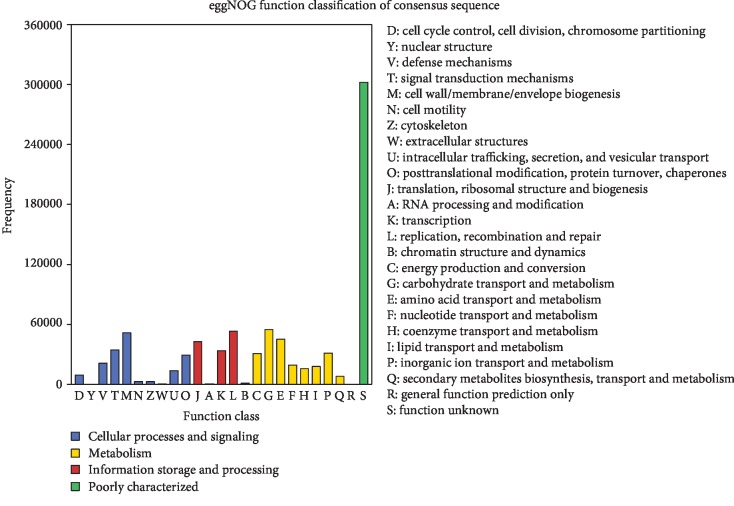
eggNOG functional annotation of identified genes.

Most of the genes identified in the rumen of cattle-yaks (54,817) were ascribed to the carbohydrate transport and metabolism category in the eggNOG database (Figure 4). In the two major categories of cellular processes and signaling and information storage and processing, the most abundant functions of the enriched genes were cell wall/membrane/envelope biogenesis (51,490 genes) and replication, recombination, and repair (53,011 genes). There were no differences between the two age groups in terms of the proportion of genes in these categories, although the number of carbohydrate transport and metabolism-related genes increased with age (*P* < 0.01) (Additional file, [Supplementary-material supplementary-material-1]). The proportion of genes classified under energy production and conversion, amino acid transport and metabolism, and inorganic transport and metabolism also increased with age, but the differences between the two groups were nonsignificant.

#### 3.3.3. KEGG Functional Annotation

A total of 234,104 genes were enriched into 369 KEGG pathways (Additional file, [Supplementary-material supplementary-material-1]). The top 10 KEGG pathways represented by these genes were related to the metabolism of small molecules—i.e., biosynthesis of amino acids, purine metabolism, carbon metabolism, and pyrimidine metabolism (Table 4). There were no differences in the proportion of genes in each pathway between age groups except for the decreased abundance of genetic information processing-related pathways and increase in metabolism pathways (Additional file, [Supplementary-material supplementary-material-1]).

## 4. Discussion

Our metagenomic analysis of rumen microorganisms of cattle-yaks of different ages revealed that bacteria were the most highly represented group (85%), with a much lower abundance of archaea. This is consistent with the previous finding that more than half of rumen microorganisms are bacteria, which play an important role in host nutrition, physiology, and immunity [10]. Notably, the archaea content in the rumen of cattle-yaks observed here was higher than that reported in an earlier study of yaks (2.27%) [11].

Rumen microbial community composition varies with the developmental stage [5]. In this study, the rumen microbe profiles of 2- and 3-year-old cattle-yaks were essentially the same at the phylum level. Firmicutes were the most highly represented phylum, followed by Fibrobacteres and Euryarchaeota. It was previously shown that these taxa are more abundant in cattle-yaks than in grazing yaks, possibly reflecting differences in adaptation to the unique plateau environment [11]. Under natural grazing conditions, yaks harbor a large number of cellulolytic bacteria such as Fibrobacter, which is the major degrader of cellulose plant biomass in the intestine of herbivores [11, 12]. The higher content of Fibrobacter in the rumen of cattle-yaks compared to yaks in the same environment indicates that the former have a greater capacity to digest fibrous material, which translates into higher production efficiency. The yak emits relatively low levels of methane, a feature that is closely related to the presence of Methanobacteria (phylum Euryarchaeota), and the Methanobacter content of cattle-yaks in this study (61%–63%) was lower than that of yaks (82.13%), indicating that the hybrid animal is superior with respect to methane emission [11].

In addition, Jami et al. also found that from the first 3 days of life to adulthood at age 2, the content of Bacteroides increased significantly with age; however, we found that although the abundance increased after 2 months of age, it remained at a certain level. This study found that at 2 and 3 years old, Bacteroidetes content was 4%. The major phyla represented in the intestine of mammals including humans and felines are Firmicutes and Bacteroides [13–16]; in the rumen of Holstein cow and buffalo, the major phylum is Bacteroides [17, 18]. In the rumen cattle-yak, Firmicutes and Fibrobacteres were the main bacteria; the relatively low Bacteroides content may be attributable to diet, species, season, and geographic location [19, 20]. Plateau yaks have a higher proportion of Firmicutes (45%) than Bacteroides (39.6%) in the intestine [21], and a similar trend has been reported in the plateau pika [22]. It was shown in ruminants that the proportion of Clostridium (phylum Firmicutes) increased with crude fiber content in hay [23]. In addition, the Firmicutes-to-Bacteroidetes (*F*/*B*) ratio was found to be higher in the rumen of cows that were fed hay as compared to grain [24]. The *F*/*B* content of cattle-yaks in this study was higher than that reported for yaks in the same environment [25]. The distribution of intestinal bacteria—especially the *F*/*B* ratio—has been linked to obesity in humans, which is thought to be related to the ability of Firmicutes to more effectively absorb calories from food [26, 27]. As an adaptation to the high-altitude environment, high intestinal Firmicutes and Fibrobacteres contents can help cattle-yaks digest crude fiber material and produce a large amount of short-chain fatty acids that are utilized by the body for energy. Many microbial species associated with lignin and cellulose degradation were present in the rumen of adult cattle-yaks including *R. flavefaciens*, *R. albus*, *F. succinogenes*, and *P. ruminicola* [28, 29], and their abundance was similar between the two age groups. Some studies have reported that *F. succinogenes* is the most highly represented of these species [17, 30], while others have shown that *R. albus* is more abundant [31, 32]. *F. succinogenes* functions in the degradation of plant cellulose [33–35]; we found here that *F. succinogenes* was the predominant species in the rumen of adult cattle-yaks whereas *R. albus* was the least abundant, which may be related to the grazing state of cattle-yaks in the plateau and long-term natural foraging behavior.

In our functional analysis of 1,281,665 nonredundant genes, 145,489 were annotated to the CAZy database. To date, 42 unique strains of cellulase- and GH-producing bacteria have been identified [36]. GHs hydrolyze glycosidic linkages between sugar molecules [3]. Most of the GHs identified in our study were oligosaccharide-degrading enzymes such as cellulase, hemicellulose, and pectinase that degrade the plant cell wall to produce oligosaccharides [37]. A higher proportion of oligosaccharide-degrading enzymes are associated with an increased rate of monosaccharide and VFA generation, which promotes nutrient absorption and utilization by ruminants [5]. In this study, the most highly represented GH family was GH2 (which comprises *β*-D-galactosidase, *β*-glucuronidase, *β*-D-mannosidase, and exo-*β*-glucosaminidase [38]) followed by the GH43 (which includes *β*-xylosidase, *β*-1,3-xylosidase, *α*-L-arabinofuranosidase, arabinanase, xylanase, galactan 1, and 3-*β*-galactosidase). Our results are in accordance with the finding that GH3 and GH2 are the predominant GHs in the rumen of Indian buffalo [3, 35, 36]. GTs catalyze the binding of sugars to glycosyl groups; we found that GT2, GT4, GT27, GT5, and GT41 families were the most highly represented in the rumen of cattle-yaks. Most of the CE and CBM genes belonged to the CE1, CE7, CE10, CBM6, CBM32, and CBM50 families and included xylan esterases of the CE1 and CE7 families, which act on branched xylan [39, 40]. We also identified 29 and 415 genes encoding cohesin and docker, respectively, which are components of the fibrosome, a multienzyme complex on the cell surface that degrades lignocellulose [41]. A total of 80 cohesin and 188 docker genes have been identified in the rumen of cows [42].

The functional annotation based on the eggNOG database revealed that the nonredundant genes in the two different age groups were mainly related to carbohydrate transport and metabolism, which could reflect the grazing environment of cattle-yaks in the plateau that requires long-term feeding in natural pastures and resistance to roughage and stress. We also found that the number of genes involved in energy production and transformation, amino acid transport and metabolism, and inorganic ion transport and metabolism was higher in 3-year-old as compared to 2-year-old cattle-yaks. Thus, the adaptability of cattle-yaks to the plateau environment increases with age. Additionally, 234,104 genes were enriched in 369 KEGG pathways, with those related to small molecule metabolism such as amino acid biosynthesis and purine, carbon, and pyrimidine metabolism being the most highly represented.

## 5. Conclusion

The results of this study demonstrate that the rumen microbiome of cattle-yaks is essentially unchanged in adulthood. Firmicutes, Fibrobacteres, Euryarchaeota, Bacteroidetes, and Proteobacteria were the predominant bacterial groups, with Firmicutes showing the highest abundance. *F. succinogenes* was the most highly represented species. An analysis of gene function found that the rumen of adult cattle-yaks is enriched in enzymes associated with lignocellulose and cellulose degradation. These results provide new insight into the heterosis of cattle-yaks and physiologic adaptations in plateau animals.

## Figures and Tables

**Table 1:** Sequence data for analyzed samples.

Sample	Raw reads	Clean reads	Cleanper (%)	Clean GC (%)	filtHost reads	filtHost GC (%)
2-year -old	102,395,772	101,320,532	98.51	42.78	84,611,980	44.03
3-year-old	83,452,932	83,117,032	99.59	48.32	81,812,068	48.53

Raw reads: No. of raw reads; clean reads: No. of clean reads; Cleanper (%): proportion of clean reads; clean GC (%): GC content of clean reads; filtHost reads: No. of filtered host reads; filtHost GC (%): GC content of filtered host reads.

**Table 2:** Assembly and nonredundant gene prediction results.

Item	Number
Number of scaffolds	1,365,143
Total length (bp)	1,396,336,337
N50 scaffold (bp)	993
N90 scaffold (bp)	556
Maximum scaffold (bp)	410,268
Mean length (bp)	1022.85
Number of genes	1,281,665
Total length	815,962,296
N50 length	708
Maximum length	26,820
Mean length	636.64

**Table 3:** Functional annotation results.

Total number of genes	1,281,665
Annotated to KEGG ortholog/number of orthologs	234,104 (18.27%)/3196
Annotated to KEGG pathway/number of pathways	234,104 (18.27%)/369
Number of genes annotated to eggNOG/number of orthologs	805,533 (62.85%)/22,766
Annotated to CAZy family	145,489 (11.35%)
Annotated to ARDB gene/number of ARDB types	275 (0.02%)/16
Number of genes annotated to VFDB/number of VFs	99,767 (7.78%)/809

ARDB: Antibiotic Resistance Genes Database; CAZy: Carbohydrate-active Enzyme; eggNOG: Evolutionary Genealogy of Genes: Nonsupervised Orthologous Groups; KEGG: Kyoto Encyclopedia of Genes and Genomes; VF: virulence factor; VFDB: virulence factor database.

**Table 4:** Top 10 enriched KEGG pathways.

Pathway	Description	Number of genes
ko01230	Biosynthesis of amino acids	26,409
ko00230	Purine metabolism	18,237
ko01200	Carbon metabolism	17,820
ko00240	Pyrimidine metabolism	16,406
ko02010	ABC transporters	14,061
ko00500	Starch and sucrose metabolism	10,978
ko00520	Amino sugar and nucleotide sugar metabolism	10,636
ko02024	Quorum sensing	10,401
ko00970	Aminoacyl-tRNA biosynthesis	10,392
ko03010	Ribosome	10,165

## Data Availability

All relevant data involved in this study are presented in the “Result” and supplementary materials.

## Supplementary Materials

Supplementary Materials Additional file: species composition. It is divided into bacteria kingdom and archaea kingdom and the composition and content of microorganisms in phylum-class-order-family-genus-species. CAZy database. GH family, GT family, CE family, and CBM family member composition and gene number. Figure S1: functional abundance of CAZy at different ages. Figure S2: functional abundance of eggNOG in different age groups. Figure S3: KEGG functional abundance in different age groups. Figure S4: KEGG pathway annotation.
