# Supplementary material for: Characteristics and Functions of the Rumen Microbial Community of Cattle-Yak at Different Ages
Source: Biomed Res Int. 2020 Mar 3;2020:3482692. doi: 10.1155/2020/3482692 (PMC7073488; doi:10.1155/2020/3482692)
Supplement: Supplementary Materials — Additional file: species composition. It is divided into bacteria kingdom and archaea kingdom and the composition and content of microorganisms in phylum-class-order-family-genus-species. CAZy database. GH family, GT family, CE family, and CBM family member composition and gene number. Figure S1: functional abundance of CAZy at different ages. Figure S2: functional abundance of eggNOG in different age groups. Figure S3: KEGG functional abundance in different age groups. Figure S4: KEGG pathway annotation. [file 3482692.f1.pdf]

Species composition

	Bacteria:85%, Archaea:15%	Bacteria: 90%, Archaea:10%
**Phylum**	**2 years**	**3 years**
Actinobacteria	0.30%	0.60%
Bacteroidetes	4%	4%
Euryarchaeota	13%	9%
Fibrobacteres	19%	23%
Firmicutes	48%	46%
Proteobacteria	1%	4%
Spirochaetes	0.90%	2%
**Class**		
Actinobacteria	0.20%	0.50%
Bacilli	0.09%	0%
Bacteroidia	3%	3%
Clostridia	41%	40%
Erysipelotrichia	0.10%	0
Fibrobacteria	16%	19%
Gammaproteobacteria	1%	3%
Methanobacteria	11%	8%
Negativicutes	0	0.03%
Spirochaetia	0.80%	1%
**Order**		
Actinomycetales	0.20%	0.30%
Aeromonadales	0.60%	2%
Bacteroidales	3%	2%
Clostridiales	34%	33%
Coriobacteriales	0.04%	0.08%
Erysipelotrichales	0.10%	0
Fibrobacterales	13%	15%
Methanobacteriales	9%	6%
Oceanospirillales	0.10%	0
others	13%	
Spirochaetales	0.60%	1%
**Family**		
Coriobacteriaceae	0.03%	0.06%
Corynebacteriaceae	0.10%	0.20%
Fibrobacteraceae	10%	11%
Halomonadaceae	0.09%	0
Lachnospiraceae	24%	23%
Methanobacteriaceae	7%	5%
others		
Prevotellaceae	2%	2%
Ruminococcaceae	3%	3%

Spirochaetaceae	0.40%	0.70%
Succinivibrionaceae	0.40%	2%

Genus

Butyrivibrio	16%	16%
Corynebacterium	0.06%	0.10%
Fibrobacter	6%	8%
Halomonas	0.06%	0
Methanobrevibacter	5%	4%
others		
Prevotella	1%	1%
Ruminococcus	2%	2%
Subdoligranulum	0.02%	0.05%
Succinivibrionaceae-uncla	0.20%	0.80%
Treponema	0.30%	0.50%

Species

Butyrivibrio-fibrisolvens	0.20%	0.06%
Butyrivibrio-unclassified	8%	8%
Corynebacterium-maris	0.03%	0.07%
Fibrobacter-succinogenes	3%	4%
Methanobrevibacter-rumina	0.30%	2%
Methanobrevibacter-unclas	2%	0
others		
Prevotella-ruminicola	0.70%	0.60%
Ruminococcus-albus	0.06%	0.07%
Ruminococcus-flavefaciens	1%	0.90%
Treponema-bryantii	0.20%	0.30%

CAZy database

GH Family		GT Family		CE Family		CBM Family	
Genes	Number	Genes	Number	Genes	Number	Genes	Number
cohesin.hmm	29	GT1.hmm	238	CE1.hmm	3018	CBM10.hmm	1
dockerin.hmm	415	GT10.hmm	54	CE10.hmm	2264	CBM11.hmm	21
GH1.hmm	160	GT101.hmm	14	CE11.hmm	171	CBM12.hmm	61
GH10.hmm	611	GT102.hmm	10	CE12.hmm	427	CBM13.hmm	215
GH100.hmm	39	GT103.hmm	2	CE13.hmm	19	CBM14.hmm	8
GH101.hmm	9	GT104.hmm	5	CE14.hmm	165	CBM16.hmm	139
GH102.hmm	4	GT11.hmm	70	CE15.hmm	327	CBM17.hmm	1
GH103.hmm	22	GT12.hmm	675	CE2.hmm	608	CBM18.hmm	1
GH105.hmm	352	GT13.hmm	153	CE3.hmm	772	CBM19.hmm	2
GH106.hmm	966	GT14.hmm	43	CE4.hmm	684	CBM2.hmm	53
GH107.hmm	5	GT17.hmm	8	CE5.hmm	4	CBM20.hmm	207
GH108.hmm	22	GT19.hmm	332	CE6.hmm	341	CBM22.hmm	151
GH109.hmm	746	GT2.hmm	4090	CE7.hmm	1093	CBM23.hmm	37
GH11.hmm	120	GT2_Cellulose_synt.hmm	8	CE8.hmm	197	CBM25.hmm	15
GH110.hmm	86	GT20.hmm	107	CE9.hmm	794	CBM26.hmm	146
GH112.hmm	14	GT21.hmm	1083			CBM28.hmm	1
GH113.hmm	84	GT22.hmm	23			CBM3.hmm	42
GH114.hmm	26	GT23.hmm	46			CBM30.hmm	69
GH115.hmm	279	GT24.hmm	45			CBM31.hmm	1
GH116.hmm	70	GT25.hmm	10			CBM32.hmm	714
GH117.hmm	712	GT26.hmm	93			CBM34.hmm	44
GH118.hmm	7	GT27.hmm	2477			CBM35.hmm	579
GH119.hmm	120	GT28.hmm	485			CBM36.hmm	179
GH120.hmm	98	GT3.hmm	275			CBM37.hmm	233
GH121.hmm	79	GT30.hmm	125			CBM38.hmm	26
GH123.hmm	91	GT31.hmm	1			CBM4.hmm	268
GH124.hmm	182	GT32.hmm	181			CBM40.hmm	92
GH125.hmm	77	GT33.hmm	74			CBM41.hmm	18
GH126.hmm	34	GT34.hmm	1			CBM42.hmm	2
GH127.hmm	410	GT35.hmm	514			CBM44.hmm	159
GH128.hmm	81	GT38.hmm	7			CBM45.hmm	13
GH129.hmm	81	GT39.hmm	158			CBM46.hmm	15
GH13.hmm	1623	GT4.hmm	2881			CBM47.hmm	81
GH13_1.hmm	1186	GT40.hmm	200			CBM48.hmm	521
GH13_10.hmm	882	GT41.hmm	2120			CBM49.hmm	10
GH13_11.hmm	797	GT44.hmm	65			CBM5.hmm	25
GH13_12.hmm	38	GT45.hmm	1471			CBM50.hmm	839
GH13_13.hmm	164	GT46.hmm	11			CBM51.hmm	80
GH13_14.hmm	199	GT49.hmm	1			CBM52.hmm	3
GH13_15.hmm	396	GT5.hmm	2325			CBM53.hmm	1
GH13_16.hmm	1070	GT50.hmm	3			CBM54.hmm	5
GH13_17.hmm	1076	GT51.hmm	480			CBM56.hmm	218
GH13_18.hmm	272	GT52.hmm	1			CBM57.hmm	25
GH13_19.hmm	1053	GT54.hmm	1			CBM58.hmm	22
GH13_2.hmm	1207	GT56.hmm	20			CBM59.hmm	2
GH13_20.hmm	1258	GT57.hmm	10			CBM6.hmm	782
GH13_21.hmm	702	GT58.hmm	2			CBM60.hmm	9
GH13_22.hmm	314	GT59.hmm	1			CBM61.hmm	185

GH13_23.hmm	974	GT6.hmm	7	CBM62.hmm	88
GH13_24.hmm	214	GT60.hmm	6	CBM63.hmm	30
GH13_25.hmm	137	GT62.hmm	20	CBM65.hmm	1
GH13_26.hmm	697	GT63.hmm	2	CBM66.hmm	97
GH13_27.hmm	527	GT64.hmm	4	CBM67.hmm	655
GH13_28.hmm	37	GT66.hmm	73	CBM69.hmm	38
GH13_29.hmm	1026	GT69.hmm	23	CBM70.hmm	18
GH13_3.hmm	362	GT7.hmm	25	CBM71.hmm	12
GH13_30.hmm	947	GT70.hmm	1	CBM72.hmm	29
GH13_31.hmm	1072	GT71.hmm	8	CBM73.hmm	5
GH13_32.hmm	670	GT73.hmm	13	CBM74.hmm	2
GH13_33.hmm	73	GT74.hmm	82	CBM75.hmm	40
GH13_34.hmm	593	GT75.hmm	4	CBM76.hmm	1
GH13_35.hmm	718	GT76.hmm	25	CBM77.hmm	13
GH13_36.hmm	1361	GT77.hmm	4	CBM78.hmm	4
GH13_37.hmm	972	GT8.hmm	263	CBM79.hmm	17
GH13_38.hmm	755	GT81.hmm	1547	CBM8.hmm	4
GH13_39.hmm	327	GT83.hmm	314	CBM80.hmm	3
GH13_4.hmm	922	GT84.hmm	22	CBM9.hmm	117
GH13_40.hmm	1049	GT87.hmm	9		
GH13_41.hmm	77	GT89.hmm	2		
GH13_42.hmm	14	GT9.hmm	171		
GH13_5.hmm	557	GT90.hmm	13		
GH13_6.hmm	532	GT92.hmm	86		
GH13_7.hmm	737	GT93.hmm	1		
GH13_8.hmm	744	GT94.hmm	175		
GH13_9.hmm	775	GT95.hmm	1		
GH130.hmm	431	GT97.hmm	1		
GH133.hmm	181	GT98.hmm	1		
GH136.hmm	62	GT99.hmm	7		
GH137.hmm	113				
GH138.hmm	59				
GH139.hmm	131				
GH14.hmm	8				
GH140.hmm	68				
GH141.hmm	224				
GH142.hmm	85				
GH143.hmm	32				
GH144.hmm	38				
GH145.hmm	13				
GH15.hmm	58				
GH16.hmm	334				
GH17.hmm	2				
GH18.hmm	204				
GH19.hmm	13				
GH2.hmm	2181				
GH20.hmm	408				
GH23.hmm	444				
GH24.hmm	85				
GH25.hmm	442				
GH26.hmm	260				

GH27.hmm	301
GH28.hmm	514
GH29.hmm	432
GH3.hmm	1347
GH30.hmm	256
GH30_1.hmm	200
GH30_2.hmm	157
GH30_3.hmm	122
GH30_4.hmm	154
GH30_5.hmm	157
GH30_6.hmm	159
GH30_7.hmm	197
GH30_8.hmm	135
GH31.hmm	896
GH32.hmm	560
GH33.hmm	323
GH35.hmm	155
GH36.hmm	697
GH37.hmm	120
GH38.hmm	75
GH39.hmm	332
GH4.hmm	105
GH42.hmm	287
GH43.hmm	1902
GH43_1.hmm	990
GH43_10.hmm	928
GH43_11.hmm	739
GH43_12.hmm	817
GH43_13.hmm	539
GH43_14.hmm	612
GH43_15.hmm	340
GH43_16.hmm	289
GH43_17.hmm	747
GH43_18.hmm	193
GH43_19.hmm	290
GH43_2.hmm	851
GH43_20.hmm	87
GH43_21.hmm	99
GH43_22.hmm	105
GH43_23.hmm	51
GH43_24.hmm	389
GH43_25.hmm	34
GH43_26.hmm	218
GH43_27.hmm	221
GH43_28.hmm	775
GH43_29.hmm	964
GH43_3.hmm	1395
GH43_30.hmm	1150
GH43_31.hmm	1217
GH43_32.hmm	730
GH43_33.hmm	1187

GH43_34.hmm	933
GH43_35.hmm	871
GH43_36.hmm	442
GH43_37.hmm	421
GH43_4.hmm	1041
GH43_5.hmm	962
GH43_6.hmm	563
GH43_7.hmm	246
GH43_8.hmm	1034
GH43_9.hmm	829
GH44.hmm	61
GH45.hmm	85
GH47.hmm	1
GH48.hmm	7
GH49.hmm	20
GH5.hmm	1553
GH5_1.hmm	364
GH5_10.hmm	58
GH5_11.hmm	46
GH5_12.hmm	155
GH5_13.hmm	61
GH5_14.hmm	205
GH5_15.hmm	174
GH5_16.hmm	28
GH5_17.hmm	14
GH5_18.hmm	35
GH5_19.hmm	540
GH5_2.hmm	163
GH5_20.hmm	45
GH5_21.hmm	139
GH5_22.hmm	635
GH5_23.hmm	38
GH5_24.hmm	106
GH5_25.hmm	748
GH5_26.hmm	408
GH5_27.hmm	212
GH5_28.hmm	304
GH5_29.hmm	389
GH5_30.hmm	56
GH5_31.hmm	27
GH5_32.hmm	44
GH5_33.hmm	14
GH5_34.hmm	18
GH5_35.hmm	90
GH5_36.hmm	507
GH5_37.hmm	622
GH5_38.hmm	490
GH5_39.hmm	626
GH5_4.hmm	664
GH5_40.hmm	268
GH5_41.hmm	44

GH5_42.hmm	152
GH5_43.hmm	39
GH5_44.hmm	177
GH5_45.hmm	159
GH5_46.hmm	657
GH5_47.hmm	49
GH5_48.hmm	256
GH5_49.hmm	96
GH5_5.hmm	553
GH5_50.hmm	155
GH5_51.hmm	93
GH5_52.hmm	463
GH5_53.hmm	52
GH5_7.hmm	226
GH5_8.hmm	14
GH5_9.hmm	277
GH50.hmm	68
GH51.hmm	685
GH53.hmm	242
GH54.hmm	35
GH55.hmm	128
GH57.hmm	204
GH59.hmm	81
GH62.hmm	4
GH63.hmm	98
GH64.hmm	13
GH65.hmm	67
GH66.hmm	24
GH67.hmm	183
GH68.hmm	5
GH70.hmm	100
GH71.hmm	4
GH72.hmm	53
GH73.hmm	227
GH74.hmm	308
GH76.hmm	217
GH77.hmm	456
GH78.hmm	970
GH79.hmm	91
GH8.hmm	214
GH81.hmm	11
GH82.hmm	44
GH84.hmm	16
GH85.hmm	17
GH86.hmm	58
GH87.hmm	96
GH88.hmm	130
GH89.hmm	138
GH9.hmm	659
GH91.hmm	7
GH92.hmm	523

GH93.hmm	210
GH94.hmm	562
GH95.hmm	502
GH96.hmm	1
GH97.hmm	724
GH98.hmm	7
GH99.hmm	122

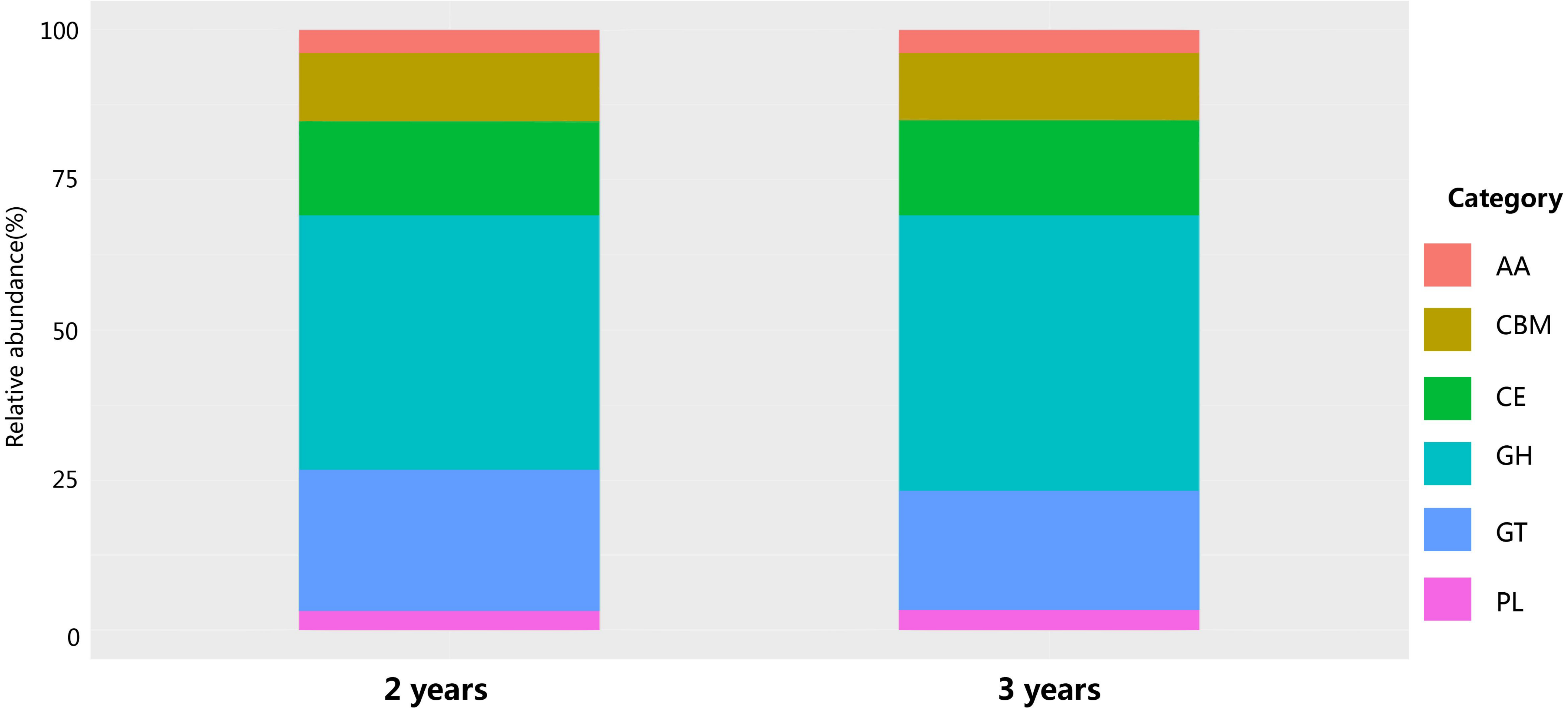

Fig S1 Functional abundance of CAZy at different ages

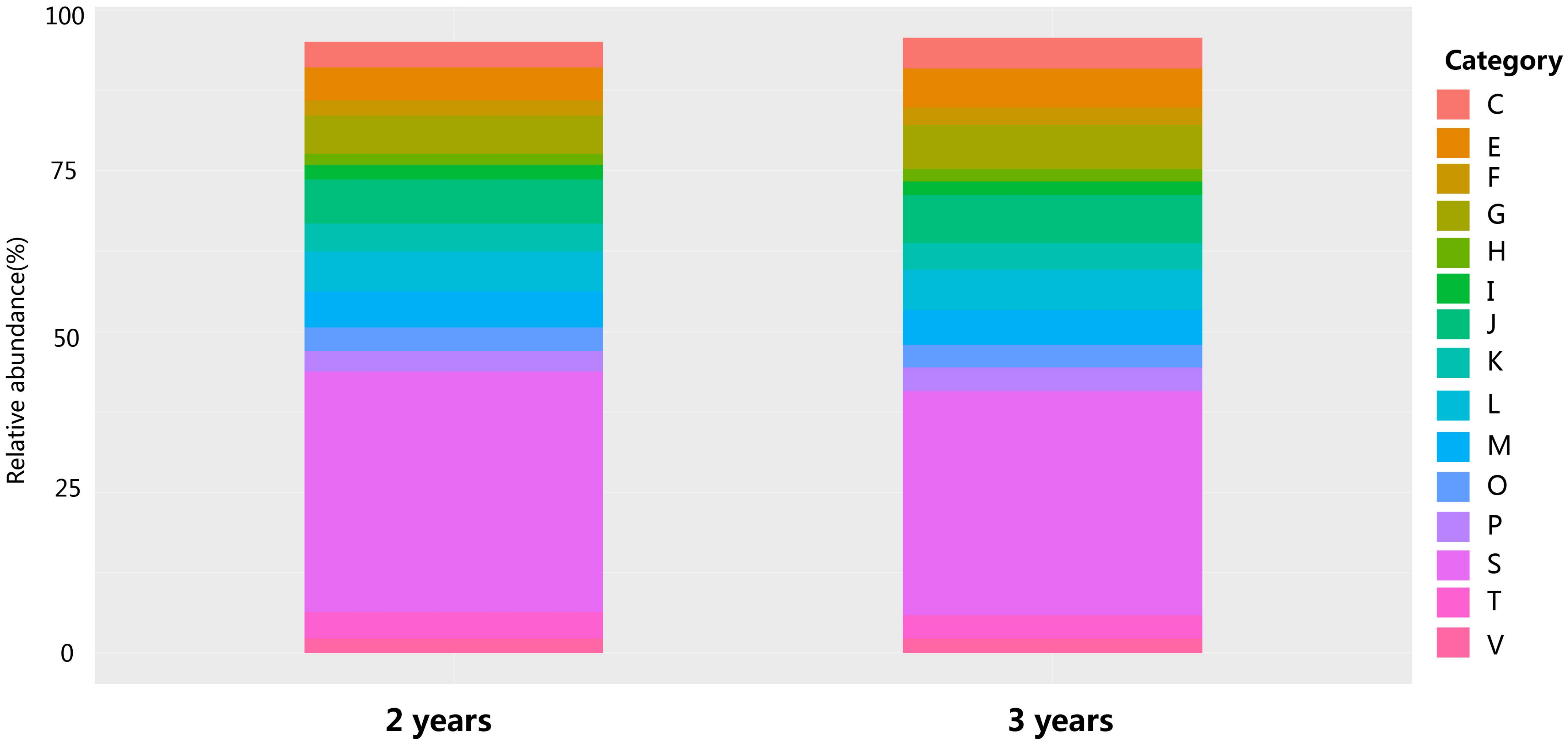

Fig S2 Functional abundance of eggNOG in different age groups

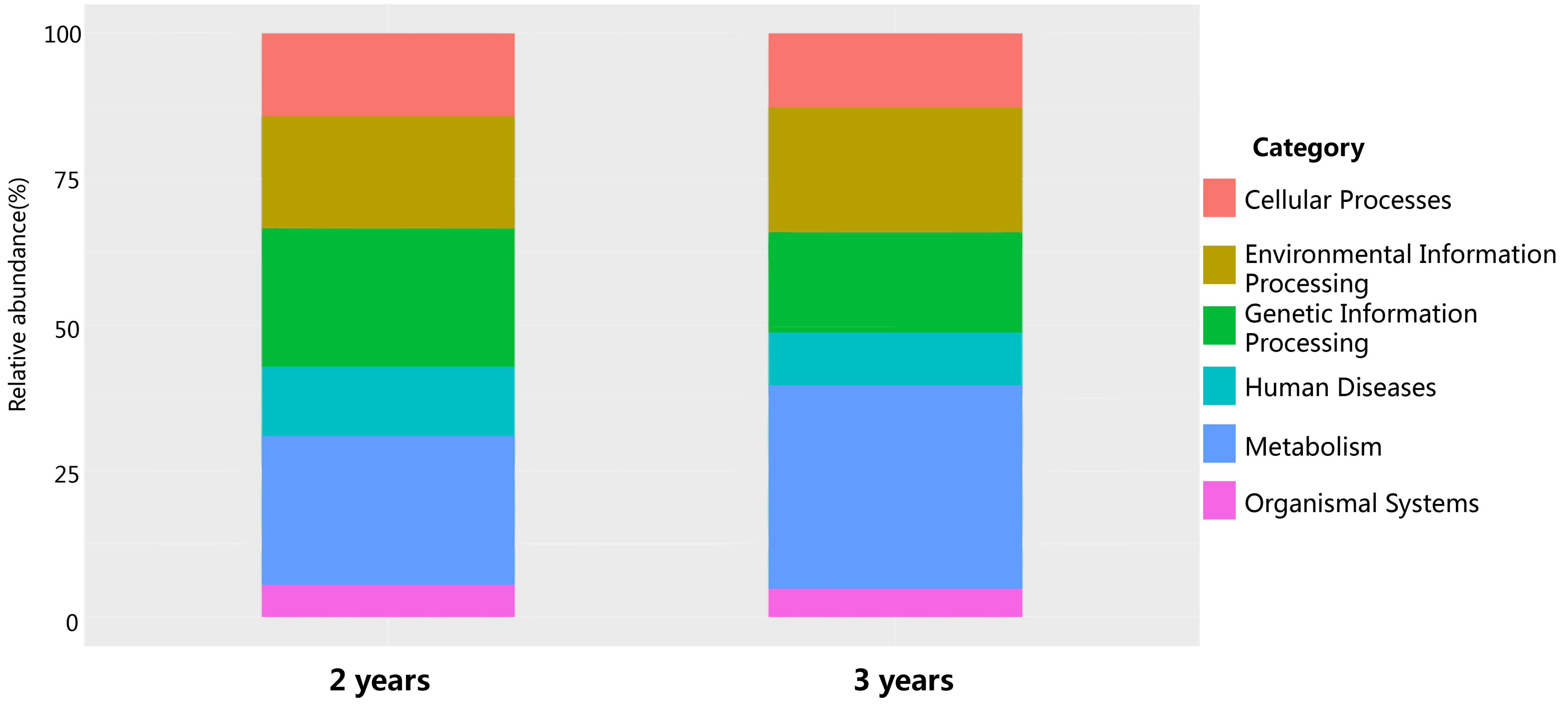

Fig S3 KEGG functional abundance in different age groups

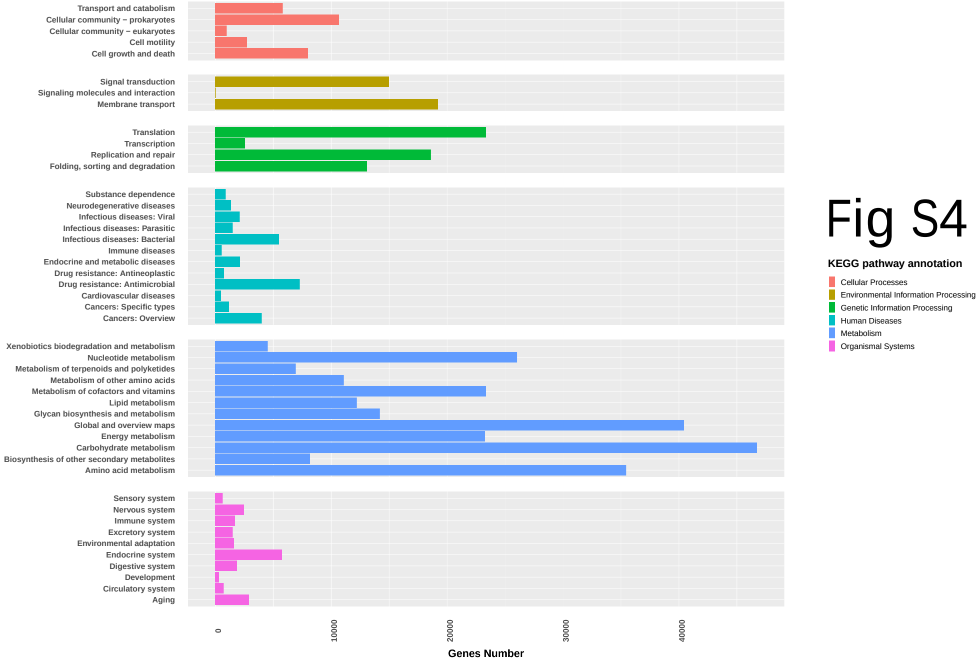

Transport and catabolism
Cellular community − prokaryotes
Cellular community − eukaryotes
Cell motility
Cell growth and death
Signal transduction
Signaling molecules and interaction
Membrane transport
Translation
Transcription
Replication and repair
Folding, sorting and degradation
Substance dependence
Neurodegenerative diseases
Infectious diseases: Viral
Infectious diseases: Parasitic
Infectious diseases: Bacterial
Immune diseases
Endocrine and metabolic diseases
Drug resistance: Antineoplastic
Drug resistance: Antimicrobial
Cardiovascular diseases
Cancers: Specific types
Cancers: Overview
Xenobiotics biodegradation and metabolism
Nucleotide metabolism
Metabolism of terpenoids and polyketides
Metabolism of other amino acids
Metabolism of cofactors and vitamins
Lipid metabolism
Glycan biosynthesis and metabolism
Global and overview maps
Energy metabolism
Carbohydrate metabolism
Biosynthesis of other secondary metabolites
Amino acid metabolism
Sensory system
Nervous system
Immune system
Excretory system
Environmental adaptation
Endocrine system
Digestive system
Development
Circulatory system
Aging
0
10000
20000
30000
40000
Genes Number
KEGG pathway annotation
Cellular Processes
Environmental Information Processing
Genetic Information Processing
Human Diseases
Metabolism
Organismal Systems

Fig S4
